# The Epic of the Thalamus in Anatomical Language

**DOI:** 10.3389/fnana.2021.744095

**Published:** 2021-10-07

**Authors:** Miguel Ángel García-Cabezas, Isabel Pérez-Santos, Carmen Cavada

**Affiliations:** Departamento de Anatomía, Histología y Neurociencia, Facultad de Medicina, Universidad Autónoma de Madrid, Madrid, Spain

**Keywords:** thalamus, Greek, Arabic, Latin, terminologia anatomica, Galen, Riolan, Willis

## Abstract

Understanding the origin of Greek and Latin words used as metaphors to label brain structures gives a unique window into how scientific and medical knowledge was produced, preserved, and transmitted through generations. The history of the term *thalamus* exemplifies the complex historical process that led to the current anatomical terminology. From its first mention by Galen of Pergamon in the 2nd century A.D. to its definitive and current use by Thomas Willis in 1664, the *thalamus* had an epical journey through 1500 years across Europe, the Middle East, and the North of Africa. The *thalamus* was confusingly described by Galen, in the Greek language, as a chamber to the brain ventricles. The term *thalamus* was transferred from Greek to Syriac through the translations of Galen’s books done in Baghdad and also from Syriac to Arabic. Then, it was translated in Europe during the Middle Ages from the Arabic versions of Galen’s books to Latin. Later, during the Early Renaissance, it was translated again to Latin directly from the Greek versions of Galen’s books. Along this epical journey through languages, the term *thalamus* switched from referring to a hollow structure connected to brain ventricles to naming a solid structure at the rostral end of the brainstem. Finally, the *thalamus* was translated from Latin to modern languages, where it is used, until today, to name a nuclear complex of subcortical gray matter in the lateral walls of the third ventricle.

## Introduction

### Metaphors in Brain Structure

Many Greek and Latin words from the *Terminologia Anatomica* used to label brain structures are descriptive and intuitive metaphors. For instance, the surface of the brain hemispheres actually looks like the *cortex* of tree trunks, the *pineal* gland has the shape of a pinecone seed, the *folia* of the cerebellum look like plant leaves, and the *olive* and *amygdala* resemble olives and almonds. Animal metaphors include *hippocampus* [seahorse] and *vermis* [worm]. The *pyramids* of the bulb, *pons* [bridge], *tectum* [roof], *septum* [wall], *fornix* [vault], *claustrum* [cloister], and *thalamus* are all architectural metaphors (FCAT, 1998).

**Figure 1:**
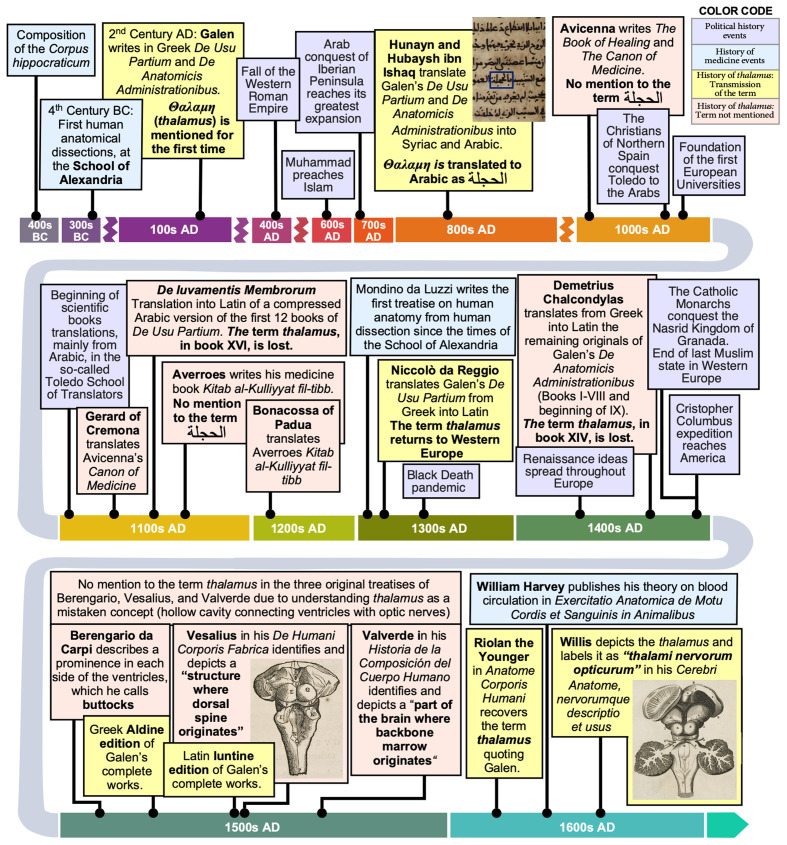
Timeline of political and scientific events related to the coining, transmission, and evolution of the term *thalamus* in neuroanatomy. Political events are shown in purple boxes. Events of the History of Medicine are shown in light blue boxes. Events related to the transmission of the term *thalamus* are shown in yellow boxes. Finally, events related to the transmission of neuroanatomical knowledge that skipped the term *thalamus* are shown in salmon boxes.

**Figure 2:**
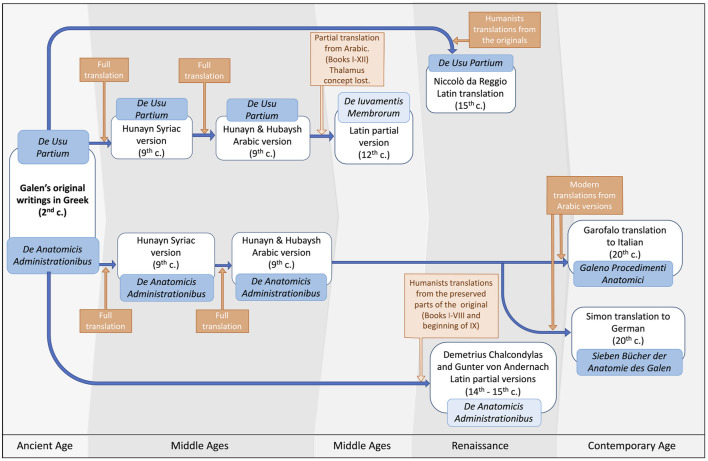
Fate of Galen’s books on anatomy. The two major books devoted to anatomy by Galen were *De Usu Partium* and *De Anatomicis Administrationibus*. These two books were translated and retranslated several times through the Middle Ages, the Renaissance, and in Modern times.

Among the Greek and Latin terms used to name brain structures, the *thalamus* stands out because even with a good knowledge of classical languages it is hard to understand its relationship with the structure that it names. The *thalamus* is an ovoid nuclear complex of subcortical gray matter located at the rostral end of the brainstem; it forms, together with the hypothalamus, the lateral walls of the third ventricle. The thalamic nuclei relay sensory information from the periphery to the cortex, participate in corticocortical communication, and connect with other telencephalic structures like the amygdala and the striatum (Jones, 2007). In many modern languages, like English or Spanish, *thalamus* means marriage bed (The Oxford English Dictionary, 1989; Diccionario de la lengua española, 2001). Why, then, use *thalamus* to name a nuclear complex—in the brain—whose shape and function do not seem related to a marriage bed? How did this term enter the anatomical language and how has it been preserved until today? In this article, we follow the use of *thalamus* as a neuroanatomical term since its first mention by Galen of Pergamon in the 2nd century A.D. to its fixation in neuroanatomical language by Riolan the Younger and Thomas Willis in the 17th century. This long trip (summarized in Figures 1 and 2) through time, space, and languages is an interesting case exemplifying the multiple influences and contributions that participate in the creation, evolution, and transmission of science and medicine, as well as their associated technical jargons.

## What Did Galen Mean by Thalamus?

Galen of Pergamon (129–199 A.D.) was an Ancient Age physician, surgeon, and philosopher. He was born in Pergamon, a Greek city under the rule of the Roman Empire, located near the coastline of the Aegean Sea, in the nowadays country of Turkey. At the age of 17, Galen started his studies in medicine in the city’s *aesculapion*, a sanctuary built in honor of Asklepios, god of healing. During the following years of his training period, he traveled and visited other Greek centers of philosophy, life sciences, and medical knowledge, such as Corinth, Smyrna, and, most importantly, Alexandria, a hub for the study of anatomy and physiology where he spent 4 years under the teachings of Marinus, an authority in anatomy and dissections. At the age of 28, Galen returned to Pergamon and started the practice of medicine, including a 5-year period as a physician to the gladiators. In A.D. 162 Galen moved to Rome, where he lived for 4 years and earned a name as a doctor due to his public demonstrations of anatomy, his success treating influential patients, and his skills in public debate. By 166, he returned to Pergamon. In A.D. 168, the Emperor, Marcus Aurelius, requested his presence in anticipation of the German tribes’ invasions; however, instead of joining the legions, Galen became a physician of the imperial court. During his stays in Rome, it is estimated that Galen wrote more than 500 treatises dealing with philosophy, science, and medicine, although approximately only one-third of them survived after a fire in the Temple of Peace in A.D. 191 (Sarton, 1954; Apuzzo, 2000; Todman, 2007).

Amongst his extensive work, Galen wrote, in Greek, several anatomy books. In these books, Galen developed the description of neuroanatomy, based on much more detailed brain dissections than anyone had done before (e.g., Herophilus). The descriptions were based mostly on animal dissections since Rome did not allow human dissections (Todman, 2007). The two most relevant Galen’s anatomy books that have survived are Π*EPI XPEIA*Σ *T*Ω*N EN AN*Θ*P*ΩΠ*OY* ΣΩ*MATI MOPI*Ω*N* [*De Usu Partium Corporis Humani* in Latin; On the usefulness of the parts of the body] and Π*EPI ANATOMIK*Ω*N E*Γ*XEIPH*ΣΩ*N* [*De Anatomicis Administrationibus* in Latin; Anatomical procedures] (Sarton, 1954; Siegel, 1968) [We shall use the Greek and Latin titles of Galen’s books throughout the text according to the canonical edition of Galen’s complete works by Kuehn (1964)]. *De Usu Partium* was written during his first stay in Rome when Galen already had a shaped view of anatomy, physiology, and healing thanks to his many years of study and his experience as a gladiator’s surgeon.* De Usu Partium* is, according to George Sarton, the most influential of all Galen’s writings, not just for its anatomical content but also as an exponent of his teleological vision of man and his parts. Also, during his first sojourn in Rome, Galen began to write *De Anatomicis Administrationibus*, but he had to rewrite it after the fire that destroyed the Temple of Peace in the capital city of the Empire. This was to be a long process that only ended shortly before his death (Garofalo, 1991). Thus, *De Anatomicis Administrationibus* is one of his most elaborated works, a product of maturity, in which he summarized all the anatomical knowledge of Greek medicine. Sarton described this book as “by far the most important of the many Galenic works devoted to anatomy” [Sarton (1954), p. 43].

Galen wrote chapters about the nervous system in both of these books, and in both of them, used the term θαλαμη (*thalamus* in the Latin translation) to name a brain structure. Book XVI of Galen’s *De Usu Partium Corporis Humani* includes in Section III the following extract:

“Accordingly, if you are willing to use a good deal of your leisure time in testing the demonstrations I have given in the thirteenth book of my treatise On Demonstrations and in certain other places to show that the instrument of vision has a luminous pneuma always flowing to it from the encephalon, you will admire the structure of the optic nerves, which have been made hollow within in order to receive the pneuma and which extend up as far as the ventricle itself of the encephalon for the same reason. For they grow out from the place where the two anterior (lateral) ventricles come to an end toward the side, and this *thalamus* itself, so to speak, of the ventricles, was made for the sake of these nerves. Anatomists have not recognized this marvelous work of Nature’s because they have not followed the ventricles to their ends, or considered for what purpose these have been so formed, or seen that the upper origins of the optic nerves are attached to the ends of the ventricles. For these reasons, then, the nerves for the eyes have been made hollow, very large, and very soft, though the other sense instruments also have large, soft nerves” [May (1968), p. 687].

Galen’s dissection manual *De Anatomicis Administrationibus* includes a description of the cranial nerves in Book XIV. In the passage where Galen describes the optic nerves, the *thalamus* is mentioned again:

*“Trovi che l’origine di ciascuno di questi due nervi tende in su all regione simile all’alcova dei due ventricoli anteriori del cervello, che si trova vicino alla sinuosità del ventricolo del cervello”* [Garofalo (1991), p. 1045].

[Find here the origin of each of these two nerves up towards the region similar to a chamber (‘*thalamus*’) of the anterior (‘lateral’) ventricles of the brain, which is found beside the sinuosity of the brain ventricle].

Is Galen describing in the above two paragraphs the ovoid nuclear complex on the walls of the third ventricle or is he referring to another structure? The term *thalamus* has four different meanings in ancient Greek. First, it means a chamber or internal room, which was generally set aside for women or for a housewife, or, at times, for a bride or for an unmarried son or daughter. Second, it has a metaphorical meaning referring to a tomb. Third, it refers to the deepest and darkest part of a ship’s hull. Finally, the term is used to refer to a certain type of shrine (Jones and McKenzie, 1996). Therefore, the term* thalamus* seems to refer to a private, hollow, isolated, architectural construction. Accordingly, in both *De Usu Partium* and *De Anatomicis Administrationibus* Galen seems to be speaking of a deep hollow brain structure connecting the optic tracts with the lateral ventricles. In fact, Arthur Earl Walker suggested that Galen’s *thalamus* referred to a part of the lateral ventricles (Walker, 1938). This idea hardly corresponds to the current understanding of a solid large ovoid mass of grey matter on the walls of the third ventricle.

To better understand Galen’s anatomical descriptions and what he possibly meant by *thalamus* it is relevant to analyze the role played by the brain ventricles in his theories on vision. Galen’s physiology was influenced by the pneumatic doctrine of Plato, Aristotle, and the Stoics. For those philosophers, pneuma, a hard-to-define ambiguous concept alluding to breath, spirit, and soul, represented both a physical agent and a force that mediated the action of organic parts. For Galen two pneumata were the agents of living functions. These pneumata were formed from inhaled air that was changed into a pneuma-like substance in the lungs and, later, into *pneuma zotikon* [vital pneuma] in the left ventricle of the heart. This “vital pneuma” reached the brain via the arterial system and was transformed into *pneuma psychikon* [cerebral pneuma] at the *rete mirabile* and the choroid plexuses. Cerebral pneuma was stored at the ventricles and then diffused through brain matter and flew through the nerves to the rest of the body (Siegel, 1968). Cerebral pneuma was necessary to mediate cerebral and psychic functions and the obstruction of its flow could produce severe disorders. For instance, in Section IX, Book IV of ΠEPI TΩN ΠEΠONΘOTΩN TOΠΩN [*De Locis Affectis* in Latin; On the affected parts], Galen proposed that obstruction of the flow of pneuma by dense humor would lead to epilepsy causing the nerves to shake to get rid of disturbing substances (Andrés Aparicio, 1997). For visual perception, cerebral pneuma needs to flow from the lateral ventricles through the optic nerves up to the eye and then back carrying the visual percept. Galen even described the optic nerves as hollow (he may have confused the central vein and artery of the retina as a central lumen of the nerve), while the rest of the nerves were solid and composed of minute fibers (Siegel, 1968). Thus, according to Galen’s physiology of vision, a connection between the ducts of the hollow optic nerves and the lateral ventricles should exist and the *thalamus* seemed to be part of this connection. In fact, Galen himself described this connection; May devoted a very long note to it in her translation of *De Usu Partium* and quoted a paragraph from ΠEPI TΩN IΠΠOKPATOYΣ KAI ΠΛATΩNOΣ ΔOΓMATΩN [*De placitis Hippocratis et Platonis* in Latin, On the Opinions of Hippocrates and Plato] where Galen wrote:

“Most anatomists know the lower orifice (of the optic nerves) at the eyes (Galen probably means the porus opticus), but nearly all of them are unaware that the upper source of these nerves is where the anterior (lateral) ventricles turn lateral … The source of the optic nerves extends to this end of the ventricles and has an orifice that is hard to see. But you will see it if you take these three things into consideration: first, the animal should be large; second, you should dissect it as soon as it has been killed; and third, the surrounding air should be clear. For if after such preparations you expose all the bodies lying upon the ventricle and remove the end of it suitably without tearing or crushing the outgrowth of the nerve, you will see the orifice at its beginning” [May (1968), p. 400].

May suggests that Galen probably had persuaded himself that he had seen what he needed to see to support his theories (May, 1968). George Sarton has suggested that Galen’s mind was “clouded by an excessive fondness for theory and classification” (Sarton, 1954, p. 79), and his insistence on the hollowness of the optic nerves and their connection with the ventricles through *thalamus* could be an illustrative example. We propose that Galen probably employed the term *thalamus* in his descriptions of the ventricles, optic nerves, and tracts to refer to a portion of the lateral ventricles, as is suggested by Hyrtl (1880) and Walker (1938), in order to stress and reinforce his pneumatic ideas on the physiology of vision.

## The Epic of Greek Science Reaching Europe Through The Arabs

After the Fall of the Western Roman Empire in A.D. 476, most Greek scientific books were lost in Western Europe. They survived, however, in the Eastern Roman Empire (Byzantium) until the Arab conquest in the 7th–8th centuries. In the 8th century, the Abbasid dynasty reached power in the Islamic world. Al-Mansur, the second Caliph of this dynasty, founded Baghdad, which soon became the Imperial capital. During the Abbasids’ rule, thanks in part to the Quran tenet of reckoning knowledge as sacred, science and philosophy were well-considered, protected, and promoted. In this context, Harun al-Rashid founded in the late 8th century the House of Wisdom in Baghdad, a translation center where both Muslim and non-Muslim scholars gathered and translated scientific and philosophic Greek works into Arabic. In the 9th century, Hunayn ibn Ishaq (809–877 A.D.), a Nestorian Christian who knew Syriac, Arabic, and Greek languages, joined the House of Wisdom as a translator and a physician himself. He translated many of the most important works by Galen and contributed to the library with some original work. He also was in charge of the House of Wisdom from A.D. 830 (O’Leary, 1949; Sarton, 1950a; Al-Khalili, 2011). The translation work in the House of Wisdom was extensive and very accurate since the translators used to collate several manuscripts to create an Arabic version of the documents (Meyerhof, 1926; Durling, 1961). The House of Wisdom “became the seed from which sprouted all the subsequent achievements of the golden age of Arabic science, from Uzbekistan in the East to Spain in the West” (Al-Khalili, 2011).

Regarding Western Europe, at the beginning of the 8th century, the Umayyad dynasty crossed the Strait of Gibraltar and soon conquered almost the whole territory of the Iberian Peninsula for the Arab Empire. In A.D. 750, the Abbasids took the rule of the Caliphate in Damascus, but Abd al-Rahman, a young Umayyad prince escaped to Al-Andalus (Muslim Spain), declared himself Emir of Cordova (the capital city of Al-Andalus), and kept conflict with the Abbasids for more than half a century. By the middle of the 9th century, peace reached Al-Andalus, and Cordova developed as a cultural hub, trying to imitate Baghdad. At the beginning of the 11th century, Cordova was the biggest, most cultured city in Europe: essential scientific volumes were preserved there. During these years, Christian reigns from the North of Spain, in turn, started the *Reconquista*, lasting from the 8th to the 15th centuries, when Christians took back the Iberian Peninsula from the Muslims (Lomba Fuentes, 1997; Al-Khalili, 2011).

In the course of the Middle Ages, scientific and cultural knowledge was transferred from the Arabs to Europe via Al-Andalus. Not only ancient Greek books translated into Arabic were re-translated to Latin and/or vernacular languages, but also new knowledge, generated by the Arabs, was transmitted to Christian Europe. This happened mainly through two processes: the emigration of Mozarabs (Iberian Christians living in territories under Islamic rule) to the North Christian Reigns of Spain; and the travels of non-Iberian Diplomatic Christians, interested in the knowledge preserved by the Arabs. These Europeans appealed to the Jews who knew the Arabic language, to translate the Arabic texts into Latin, the European scholar language at the time (Lomba Fuentes, 1997; Al-Khalili, 2011). After the conquest of Toledo in 1085 by the Christian King Alfonso VI, a translation initiative took place in this city in the center of Iberia, where many Jews arrived fleeing from the religious intransigence of the Almohad Caliphate ruling Al-Andalus by that time. The translation work resulted in what is known as the “Toledo School of Translators”, where many important ancient philosophical, scientific, and medical books, including those of Galen, were translated from their Arab versions to Latin and were made available to European scholars. The Crusaders during the 12th century also brought back to Europe abundant Arab manuscripts of ancient Greek books (O’Leary, 1949; Sarton, 1950a; Lomba Fuentes, 1997; Al-Khalili, 2011).

The development of the Universities in Western Europe in the 12th–13th centuries facilitated the spread and the evolution of the recently re-incorporated Greek knowledge, and the development of modern science during the Renaissance. During the Renaissance period, another episode of the epic of Greek science reaching Europe took place: Renaissance was a period of rediscovery of the Greek language, starting in Italy. During those years, Western European scholars demonstrated an interest to approach the original Greek philosophy, literature, science, and medicine. Galen’s works, which had been previously translated from Arabic versions to Latin and included in the Universities *curricula*, were re-translated and commented on by Renaissance humanists who knew Greek and were able to read Galen “in the original”. These new translations were usually complemented with comments from the translators themselves to facilitate the comprehension by students with poor or null knowledge of Greek (Bacalexi, 2017; Bernard-Pradelle, 2020). Figure 1 summarizes the historical events related to the transmission of Greek science, and of Galen’s work, to Western Europe.

As explained further below, Galen’s book *De Usu Partium* followed the complex pre-Renaissance journey of ancient science: it was first translated to Syriac and then to Arabic in the House of Wisdom in Baghdad; then, it was translated to Latin from a shortened Arabic version by the Toledo School of Translators. This shortened Arabic version did not include the part where the *thalamus* is mentioned, and therefore the concept of *thalamus* did not return to Europe until the late Middle Ages/early Renaissance when all the books in *De Usu Partium* were translated from the original Greek into Latin. With respect to *De Anatomicis Administrationibus*, it was translated to Arabic in Baghdad, but it was never translated from Arabic to Latin in Western Europe during the Middle Ages, and the parts of the original Greek version that contained the reference to the *thalamus* were permanently lost, so this part of *De Anatomicis Administrationibus* was available in Europe much later than *De Usu Partium* (Simon, 1906). Figure 2 summarizes the fate of *De Usu Partium* and *De Anatomicis Administrationibus* and their translations from their writing to the 20th century.

## The Galenic Concept of Thalamus in Islamic Medicine

*De Anatomicis Administrationibus* and *De Usu Partium*, like many other works by Galen, were translated into Syriac by Hunayn ibn Ishaq at the House of Wisdom in Baghdad. Both books were then translated into Arabic by Hubaysh, a nephew of Hunayn, probably under the supervision of his uncle (Bergsträsser, 1925; Meyerhof, 1926; O’Leary, 1949).

**Figure 3:**
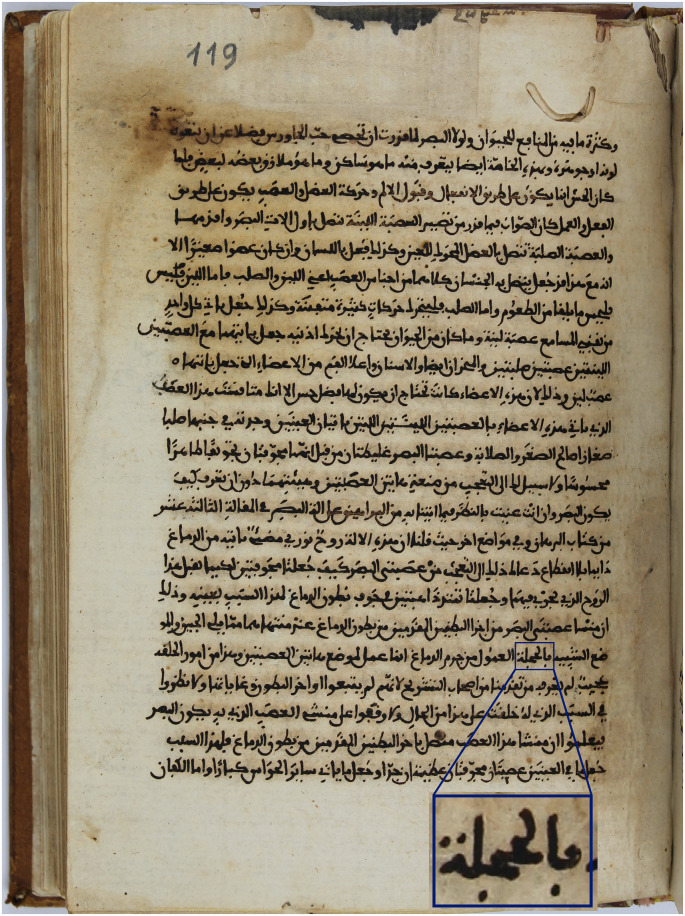
Page 119 of the manuscript of the Arabic translation of *De Usu Partium* (ibn Ishaq, 809–877 A.D.) preserved in the Real Biblioteca del Monasterio de El Escorial, Spain. The inset highlights the word 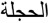 used by the translator to refer to *thalamus*.

We have looked at the description of the *thalamus* in Book XVI of the Arabic manuscript of *De Usu Partium* at the *Real Biblioteca del Monasterio de El Escorial* [Royal Library of El Escorial Monastery] in Spain: the Arabic translator wrote “a location similar to “
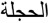
” [ibn Ishaq (850) p. 119] (Figure 3). This word (“
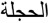
”) has several meanings in Arabic, the first of which refers to a canopy or alcove for a bride in a house (Lane, 1968), just as *thalamus* in classical Greek. Thus, in our opinion, the Arabic translators correctly grasped the Galenic concept of the *thalamus* and made a proper translation: a hollow structure connecting the optic nerves with the lateral ventricles that was like a curtained canopy or chamber for a bride.

The Arabic translations put the pneumatic theories of Galen in the hands of the Arab physicians, who did not perform anatomical dissection and mostly relied on Galen for anatomical and physiological knowledge (Campbell, 1926; O’Leary, 1949). Avicenna (980–1037), the most relevant and influential Arab physician, in Book I of his *Liber canonis medicinae* [The canon of medicine], described the origin of the optic nerves at the end of the lateral ventricles, but he did not mention the hollowness of these nerves. In Book III, Avicenna described the brain and its ventricles and attributed to each of them a function related to the production and circulation of cerebral pneuma. There is also a confusing paragraph where Avicenna described a worm-like structure joined to two oblong eminences that can close and open the passage of pneuma through the third ventricle (de Koning, 1903/1986). According to Hyrtl (1880), these eminences were the thalami, but Pieter de Koning considered them to be the superior colliculi, named “buttocks” by Galen (de Koning, 1903/1986). Averroes (1128–1198), another Arab physician, in the Book on the Anatomy of Members in his *Kitab al-Kulliyyat fil-tibb* [Book on Medical Generalities] (Vázquez de Benito and Álvarez Morales, 2003), described the optic nerves as hollow, mentioned the *rete mirabile* and described the brain ventricles in a similar way to Avicenna:

*“En su interior (‘de la cabeza’) tiene cavidades que se comunican unas con otras y se llaman ventrículos del cerebro. Dos de ellos están en la parte anterior de éste, uno en el centro y otro en la parte posterior. En el lugar en donde se unen estos ventrículos hay unos cuerpos que están conformados de modo que puedan cerrarse en unos momentos o abrirse en otros”* [Vázquez de Benito and Álvarez Morales (2003), p. 58].

[Inside (‘the head’) there are inter-connecting cavities named brain ventricles. Two of them are in the anterior part of the brain, one is in the middle, and another in the posterior part. At the place where these ventricles join there are some bodies that are so built that they can open or close].

Neither Avicenna nor Averroes describe a hollow structure connecting the ventricles and the optic nerves and they do not explicitly mention the “
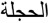
” of the Arabic version of *De Usu Partium*. However, Galen’s ideas on pneumatic and ventricular doctrine were adopted by the Arab physicians and formed the basis of the physiological system of Arabic medicine during the Middle Ages.

In sum, the translation of the term *thalamus* from Greek to Arabic did not lead to any further development of this concept by the Arab physicians.

## Middle Ages in Europe: No Trace of The Galenic Hollow Thalamus Or The Canopy of The Arabic Translations

It is well known that Galen’s views on anatomy and physiology reached the European Universities from the 12th century onward as Latin translations from both the books of Arabic physicians and from the Arabic versions of Galen’s books. For instance, Gerard of Cremona (1114–c. 1187) translated Avicenna’s Canon in Toledo, and Bonacossa of Padua translated Averroes’ *Kitab al-Kulliyyat fil-tibb* in 1255 (Campbell, 1926; Sarton, 1950b). The first 12 books of *De Usu Partium* were also translated from Arabic into Latin during the 12th century, probably from an already-compressed Arabic version; this abridgment was known as *De Iuvamentis Membrorum* [On the use of members] and it spread all over Europe as the only treatise on human anatomy in Latin available for surgeons and physicians until the 14th century (French, 1979). However, the Arabic version of* De Anatomicis Administrationibus* was not translated into Latin during the Middle Ages, although it was cited as *De Iudicacione Anathomie* [On the knowledge of anatomy] by several Christian physicians like the Spaniard Arnau de Vilanova (1240–c. 1311) or the French Guy de Chauliac (1260–c. 1368), who knew about it by indirect mentions (García-Ballester, 1982).

The Latin translations from the Arabic versions of Galen’s works and from the books of Arab physicians themselves, who were Galenic in their anatomy and physiology, allowed a complete knowledge of Galen’s physiology, pathology, and therapeutics and gave rise to what García-Ballester called the “New Galen”, a collection of Latin translations from Arab versions of Galen’s books that was introduced in the curriculum of many European Universities at the beginning of the 14th century (García-Ballester, 1982). Mondino da Luzzi (1270–c. 1326), a University teacher in Bologna who was influenced by the “Arabized Galenism” of the “New Galen”, wrote the first treatise on human anatomy from human dissection since the times of the School of Alexandria (Singer, 1957). In the introduction of his “Anathomia” he quoted *De Iuvamentis Membrorum* several times and also recognized the authority of Avicenna and Averroes (Wickersheimer, 1926). He described the structure, function, and diseases of brain ventricles in this way:

*… “si sit oppilans ex toto aut oppilat ventriculos et substantia simul aut ventriculos tantum. Si ventriculos et substantiam sic est apoplesia. Si ventriculos tantum sic est epilesia. Verum est ¿? hec oppilatio est oppilans. Ventriculos alios sive occupans licet non tantum quantum anteriorem. … ante aut quam procedas ad ventricum medium considra intermedia inter hunc et medium et sunt tria anche qui sunt sicut basis buius modi anterioris ventriculi dextri et sinistri et substantia cerebri ad formam et figuram anchass et a latere uni cuiusquem intra ventriculos iam dictos substantia una ¿? sanguinea facta ad modum vermis oblongi sive subterranei ligata ligamentis et nervulis alterutrinque que ad sui elongationem constringit et claudit anchas et via sive transitu ab anteriori ad medium et econtra et quando homo vuit cessare a sui cogitatione et consideratione elevat parietes et dilat anchas ut posit spiritus transire ex uno ventriculos ad alios”* [Wickersheimer (1926), p. 43].

[… if there is a complete obstruction it can obstruct the ventricles and the brain matter at once or only the ventricles; if it is ventricles and brain matter, then it is apoplexy, if only the ventricles, then epilepsy. This obstruction can affect the other ventricles, although not as much as the anterior (‘lateral’) ventricle. … but before you proceed through the middle ventricle take into account the intermediate part between this (‘the lateral ventricle’) and that in the middle (‘third ventricle’). There are three things: the *anchae* that are like the base of the anterior ventricle on the left and right. They are made of brain matter and have the shape of *anchae* (‘buttocks’). Beside each of them, between the aforementioned ventricles, there is a bloodish red substance formed like a long worm, like an earthworm, attached by nerves and ligaments on each side that by narrowing its length closes the *anchae* and the passage from the anterior to the middle ventricle and the reverse. When a man wishes to stop thinking and reflecting he raises the walls and dilates the *anchae* so that the spirit (‘pneuma’) may pass from one ventricle to the others].

The Galenic and Arabic influences are clear. Is Mondino referring to the *thalamus* when he uses *anchae* [buttocks]? Walker thought so (Walker, 1938), but Edward Jones considered the term *anchae* equivocal (Jones, 2007). Later in the text, Mondino described the optic nerves and stated that they were in continuity with brain matter and with the anterior ventricles. Thus, it seems that he assumed the pneumatic theory of vision from Galen, but he did not use the term *thalamus* or canopy, probably because he did not have Latin or Arab versions of either Book XVI of *De Usu Partium* or Book XIV of *De Anatomicis Administrationibus* to hand.

## Early Renaissance: The Galenic Hollow Thalamus Was Translated to Latin from Galen’s Originals, but It Is Not Found in Pre-Vesalian and Vesalian Anatomy

During the 14th century several of Galen’s manuscripts were translated directly from Greek into Latin (Figures 1 and 2). These direct translations and the Greek manuscripts progressively substituted the “New Galen” Latin translations and were the basis for the printed humanist editions of the first half of the 16th century: The Greek Aldine edition (Galen, 1525) and the Latin Iuntine edition (Galen, 1541). These editions made Galen’s original works accessible to the Renaissance anatomists (Durling, 1961; Fortuna, 2005).

All the books in *De Usu Partium*, including Book XVI where the *thalamus* was mentioned, were translated into Latin by Niccolò da Reggio (c. 1280–c. 1350) shortly after Mondino’s Anatomy was written (Sarton, 1950c; McVaugh, 2006). However, *De Anatomicis Administrationibus*, whose Arabic version was not translated into Latin during the Middle Ages, underwent a different fate: only the first eight books and the beginning of the ninth were available in the original Greek during the Renaissance and were translated into Latin, first by Demetrius Chalcondylas (1423–1511) and later by Günter von Andernach (1505–1574) (Fortuna, 1999). Most of Book IX and Books X to XV, including Book XIV where the *thalamus* was mentioned, were only preserved in the Arabic version. These were made available to the Western world much later through their translation from Arabic into German by Max Simon at the beginning of the 20th century (Simon, 1906).

The most relevant anatomist of the early Renaissance was Jacopo Berengario da Carpi (1460-c. 1530). He had access to Galen’s works printed by the humanists; in fact, he revised and published the translation by Demetrius Chalcondylas of the first nine books of *De Anatomicis Administrationibus* (Fortuna, 1999), but he still relied mostly on Mondino and the Arab physicians. His description of the brain was an advance over Mondino (Singer, 1957), especially regarding the general form of the ventricles:

“In the ventricle on both sides near the base is a peculiar red substance called a worm, composed of veins and arteries, which reaches from one end to the other of each ventricle. This has motion, according to some, opening and closing the ventricles voluntarily.

Below the worms at their sides is a certain eminent part of the brain, which many compare to the human buttocks in its form. This part both in elongation and closing of the ventricles touches its two portions together and separates them in the shortening and dilation of the ventricles” [Lind (1959), p. 143].

According to Paul G. Roofe, the “buttocks” mentioned by Berengario da Carpi could refer to the head of the caudate nuclei (Lind, 1959). Later on, Berengario described the third ventricle:

“Near this *embotum* toward the rear also under the ventricles mentioned before is a certain somewhat oblong vacuity whose walls are like the aforesaid buttocks. These close and open this vacuity when there is need either by the motion of the aforesaid worms which are immediately above them or by another motion caused by the spirits. Authors commonly regard this vacuity as the middle (third) ventricle, in which they say there exists the cogitative or reasoning faculty” [Lind (1959), p. 144].

After Avicenna’s eminences, Averroes’ bodies and Mondino’s *anchae*, Berengario’s buttocks *in the walls of the vacuity that some authors regard as the middle ventricle* could actually be the current hemithalami. But why, if he had the complete Latin translation of *De Usu Partium* to hand, did he not mention the *thalamus*? Berengario was clearly influenced by Mondino and the Arabs, but his work already contains some criticism; for instance, he denied the existence of the *rete mirabile* and stated that although some anatomists describe the optic nerves as hollow, this is not visible in the dead creature (Lind, 1959). Thus, one can speculate that if the optic nerves are solid, then Berengario did not need a *thalamus* to connect them with the ventricles.

Andreas Vesalius (1514–1564) also had access to the humanist editions of the Galenic corpus. In fact, his first work, still as a student, was helping his mentor Johannes Günter von Andernach, who had previously translated *De Anatomicis Administrationibus*, in the production of a textbook on Galenic anatomy for students (Singer, 1957; Fortuna, 1999). Vesalius was very influenced by Galen’s views on physiology, but in his *De Humani Corporis Fabrica* [The factory of the human body] (Vesalius, 1543/1997) he began systematically to point out the mistakes in Galenic anatomy. Nancy G. Siraisi states that “his attitude to Galen, his principal ancient predecessor, was a mixture of dependence, reworking, and critique” [Siraisi (1997), p. 2]. In the *Fabrica* Vesalius cited *De Usu Partium* several times, but *thalamus* does not appear in the book and, in describing the ventricular system, he denied the existence of continuity between the brain ventricles and the optic tracts and nerves:

*“Haec quum dico, innunera Galeni dogmata mihi reclamare haud ambigo, quae olfactus organa cerebri anteriores esse ventriculos variè docent, et eosdem ventriculos tenues sensim factos in visorios nervos suo mucrone finire”* [Vesalius (1543/1997), p. 786].

[In saying such a thing, I am aware that I am challenging innumerable theories by Galen, who stated that the anterior (‘lateral’) brain ventricles were the smell organs and that such ventricles became thinner and thinner and, finally, they ended at the optic nerve].

**Figure 4:**
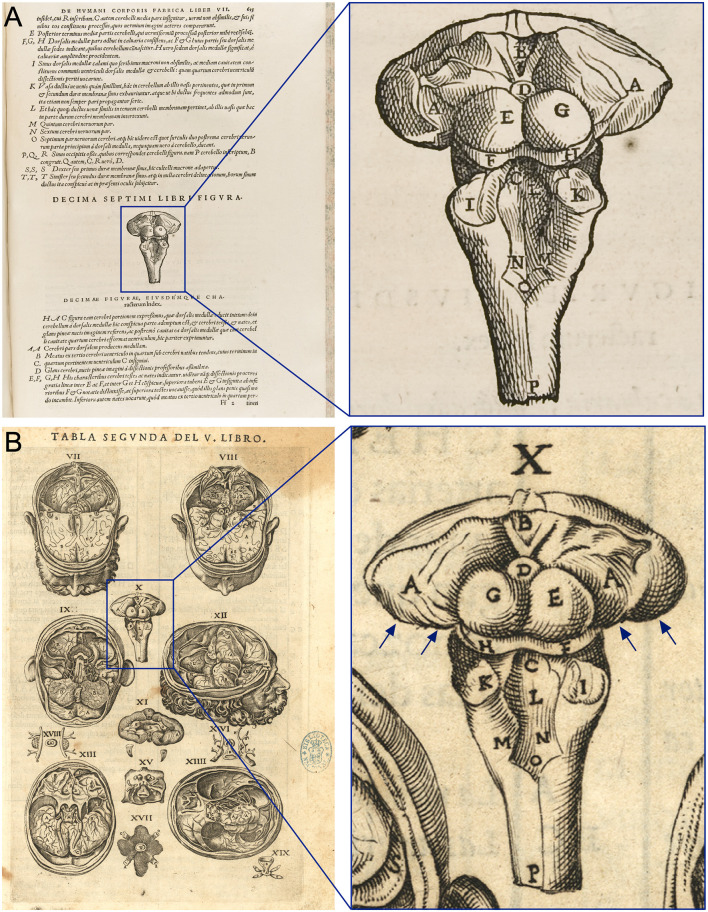
Vesalius’ and Valverde’s drawings of the posterior view of the human brainstem and deep gray matter of the brain.** (A)** Tenth Figure of Book VII of *De Humani Corporis Fabrica* (Vesalius, 1543) where Vesalius identified the *thalamus* (A in Vesalius’ figure) as “*Cerebri pars dorsalem producens medullam”* (dorsal part of the brain that originates the dorsal cord). **(B)** Second Table of Book V of *Historia de la composición del cuerpo humano* (Valverde de Hamusco, 1556) -left- with the figure X of this table expanded to the right. In this figure, Valverde identified the *thalamus* as “*la* *parte de los sesos de que nace el tuétano del espinazo”* (the part of the brain from which the backbone marrow originates). He also draws for the first time the geniculate nuclei of the thalamus (blue arrows).

Vesalius also stated that the optic nerves are not hollow. Thus, as for Berengario, there was no place for the *thalamus* in Vesalius’ anatomy. Even more, for Vesalius, the ventricles lacked any function related to the faculties of the soul in contrast to Mondino and Berengario, despite his notion that they produced the animal spirits that were distributed by the nerves to the organs of sense and motion. Interestingly, Vesalius was the first to draw the brain structure that we currently call the *thalamus*: in the 10th Figure of Book VII of the *Fabrica*, the author shows that the dorsal cord (which, according to Vesalius, included the spinal cord and the current brain stem) originates in the structure we call *thalamus* nowadays (Figure 4A). In the text, Vesalius described the masses of deep grey matter that constitute the striatum, the diencephalon, and the midbrain.

“*Praeterea in basi eas quoq; esse continuas ostendit, cum magna & fecundùm longitudinem & profunditatem qua continuantur regio, tum cerebri testes & nates, omniumque; maximè ex cerebri basis medio pronascens dorsalis medullae initium, quod amplum crassumque; est, & perinde ac illae duae cerebri partes hoc in loco continuum unumque”* [Vesalius (1543/1997), p. 779].

[Moreover, at the base (‘of the brain’) it is seen that, on the one hand, the great region along which the two parts of the brain have continuity, whether in length or in depth, and, on the other, the brain testes and buttocks also have a continuity; and, more importantly, the origin of the dorsal spine that is located in the middle of the brain base, from a place that is wide and thick, also has continuity as in the case of the two parts of the brain].

Juan Valverde, the most prominent Spanish anatomist in the 16th century, did not mention the *thalamus* but, like Vesalius, draw the hemithalami in the 10th Figure of Book V of his *Historia de la composición del cuerpo humano* [History of the composition of the human body] as “*la* *parte de los sesos de que nace el tuétano del espinazo”* [the part of the brain from which the backbone marrow originates] [Valverde de Hamusco (1556), p. 82–83], so Valverde’s backbone marrow would be the equivalent of Vesalius’ dorsal cord (Figure 4B). In his Figure X, Valverde also depicted the geniculate nuclei for the first time (Valverde de Hamusco, 1556).

According to Walker (1938), the Renaissance anatomists did not pay much attention to the *thalamus*, but as we have noted above this is not quite so. Berengario very probably referred to both hemithalami as “buttocks” and Vesalius and Valverde clearly depicted them as oval masses in the figures of their anatomy books. All of them knew the anatomical works of Galen from the printed humanist editions and probably had read the paragraph of *De Usu Partium* describing the *thalamus*. But, for these anatomists, the term *thalamus* referred to a concept related to an anatomical detail, the hollowness of the optic nerves, which they rejected based on their own observations while dissecting cadavers; consequently, they did not use *thalamus* in their descriptions.

## Early 17th Century: Jean Riolan’s Devotion to Galen Brought The Galenic Thalamus Back to Anatomical Terminology

Jean Riolan the Younger (1580–1657) was the first anatomist to cite the paragraph of *De Usu Partium* in which Galen described the *thalamus*. Riolan was a scholar and a humanist fascinated by Ancient Greece and Rome, a fully convinced Galenist who considered Galen the most skilled anatomist of all times. He was also a strong opponent to William Harvey (1578–1657) and his theory on the circulation of the blood (Mani, 1968). Riolan’s first anatomical work was *Anatome Corporis Humani* [Anatomy of the human body], published inside the *Opera Omnia* of his father (Riolan, 1610). When describing the optic nerves, Riolan the Younger cited Galen:

*“Itaq; prima Conjugatio erit nervorum Opticorum, qui à posteriore cerebro prodeuntes, ex sententia recentiorum Anatomicorum, per calvariae foramen in oculi centru definunt. Gal. à natibus cerebri nervos opticos derivat, istasque eminentias θαλαμoζvocat, in gratiam nervorum opticorum constructas. Opus naturae admirabile Anatomicis incognitum lib. 16. de usu part. ca. 3”* [Riolan (1610), p. 111].

[Thus, the first pair of nerves is the optic nerve pair that, coming from the posterior brain, in the opinion of modern anatomists, passes to the center of the eye through an opening of the skull. Galen states that the optic nerves originate in the buttocks of the brain, eminences that he calls θαλαμoζcreated for the sake of the optic nerves. An admirable work of nature unknown to anatomists, XVI Book, De Usu Partium, section 3].

*“Ad spiritus animalis delationem Opticos perforatos esse scribit Galenus lib. 10. de usu part. Sed nulla cavitas recentioribus Anatomicis diligenter intuétibus apparuit. Ipsemet Vesalius cum summam operam adhibuitsset in peruestiganda optici nervi cavitate, tandem deprehendit solidum esse ex varijs fibris conflatum, interveniente passim substátia medullari. Qui verò nervi optici cavitatem defendunt in simijs manifestam esse dicunt: Quinetiam in homine modo Nervus ab exortu non statim transversim fecetur, sed paulò ante insertionem, Verum Gal. nulla eget excusatione & defensione, cum ipse suam opinioné interpretetur lib. 7 de Plat. & Hipp. c.4. & libello de ocul. c.3. Tres enim conditiones requirit ad pervidendum optici foramen ut animal sit magnum, nuper mactatum, & in lucido aëre conspiciatur”* [Riolan (1610), p. 112].

[Galen, in Book 10 of *De Usu Partium* describes the optic nerves as hollow so as to allow the diffusion of the animal spirits. Anyway, no cavity is patent to modern anatomists, regardless of a careful search for it. Vesalius himself, in spite of devoting great efforts to the investigation of the hollowness of the optic nerve, finally concluded that it was a solid composed of several fibers made of an orderless medullary substance. Those who truly defend the hollowness of the optic nerve state that it is manifest in simians: however, in Man, the nerve would be discontinuous and transverse from its beginning, to shortly before its insertion, but Galen lacks an excuse or defense when he himself will comment his opinion in Book 7 of On the Opinions of Hippocrates and Plato (c.4) and in his opuscule On the Eyes (c.3). In fact, three conditions are required to clearly see the opening of the optic nerve: the animal must be large, it must have been sacrificed recently and it must be examined attentively in a clear atmosphere].

Thus, Riolan, a lover of ancient medicine, came into conflict with the modern research of his time. In another passage, Riolan gave an explanation on why such terms like “*thalamus”*, “*nates*” (buttocks), and “*testes*” (testicles), were used by anatomists:

*“At secundum tertis ventriculi ductum plures particulas notabis, quibus verteres Anatomici obscoenarum partium nomina propter similitudem & ad distinctionem indiderunt. Primae & maiores eminentiae quae posteriors fornicis columnas amplexantur thalami nervorum opticorum dicuntur a Galeno, quos in gratiam eorum nervorum constructos esse statuit. Sequentes duae eminentiae testes vocantur, quibus aliae duae subiectae nates appellantur, earum fissura anus”* [Riolan (1610), p. 159–160].

[Passing from the second to the third ventricle you will observe three small parts named by ancient anatomists after the names of obscene parts because of their resemblance and to make them distinguishable. The first and larger reliefs that embrace the posterior columns of the fornix are named by Galen thalami of the optic nerves; he stated that they were made for the sake and protection of these nerves. The next two eminences are named testes, the two others placed below them are named buttocks, between them lies the gluteal sulcus].

Riolan was a very popular and influential teacher; his anatomy books were the most read during the 17th century, together with those of Bartholin, and he introduced many human anatomy terms (Singer, 1957). Had Riolan used a term other than *thalamus*, we would probably call it differently nowadays.

## Late 17th Century: Thomas Willis Fixed The Term Thalamus in Brain Anatomy

A few years after Riolan’s death, Thomas Willis (1621–1675) flourished as the most prominent brain expert of the 17th century. His *Cerebri Anatome, nervorumque descriptio et usus* [The anatomy of the brain and nerves], which was published in 1664 and translated into English in 1681 (Willis, 1664, 1681/1978), can be considered the pioneering work for modern Neuroanatomy and Neurology (Hughes, 2009). Willis divided the central nervous system into the brain, oblong marrow or brain stem, cerebellum, and spinal marrow. According to Willis, the oblong marrow consisted of several portions beginning with the striatum and ending at the spinal cord:

“We will pass now from the brain to the explication of its Trunk, to which both it and the Cerebel do grow like mushrooms or large excrescences. This part is commonly called the Oblong Marrow; under which name we comprehend all that substance which reaches from the inmost Cavity of the Callosus Body, and conjuncture in the basis of the Head, to the hole of the hinder part of the Head; where the same substance, being yet far continued, ends in the Spinal Marrow” [Willis (1681/1978), p. 101].

Willis stated that the brain stem is Y-shaped and is divided into different portions, of which those most anteriorly located are the striatum. Where the striatum ends:

… “a marrowy substance succeeds, which being somewhat of a darkish color going forward for some space, is distinguished by a peculiar bending forward from the other contiguous parts. This Galen (perhaps not improperly) calls the Chambers of the Optick Nerves [thalami nervorum opticorum in the Latin version]; for in this place the Optick Nerves shewing themselves from the highest region of either side, being carried downward with a certain compass, are united about the Tunnel” [Willis (1681/1978), p. 103].

**Figure 5:**
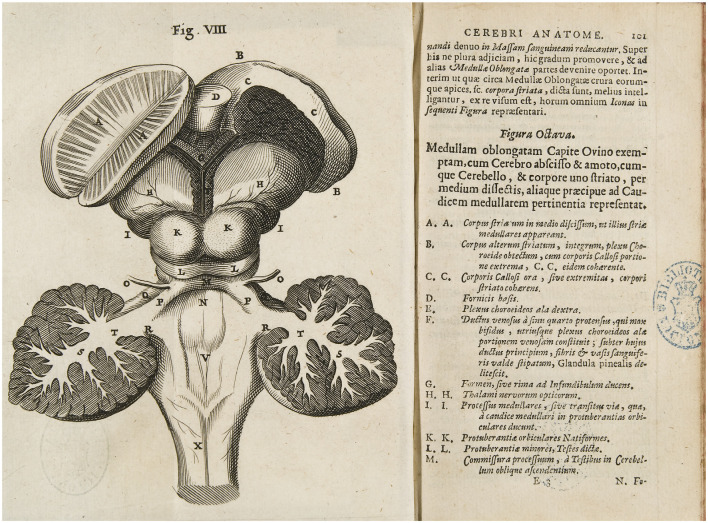
Willis drawing of the posterior view of the brainstem and deep gray matter of the human brain. Figure VIII of *Cerebri Anatome, nervorumque descriptio et usus* (Willis, 1664), where Willis used *Thalami nervorum opticorum* to identify the *thalamus* (H), and also draws the geniculate nuclei *of the thalamus* (I), which he identified as *Processus medullares*.

In Figure VIII of *Cerebri Anatome*, the *thalamus* is depicted quite similarly to the Vesalius and Valverde figures discussed above (Figure 5, compare with Figure 4). What, for these authors, was the part of the brain constituting the origin of the dorsal spine is, for Willis, a portion of the oblong marrow where the optic nerves originate; thus, Willis named them thalami. One can wonder why the British Willis used the term *thalamus*, a term recovered from Galen by Riolan, the most prominent rival of Harvey. We can only speculate. Willis was a very conservative man and was still strongly influenced by Galen’s physiology: he still believed in the fluid of vital spirits and their role in brain function, even if there were some doubts in his mind, because he admitted that when cutting nerves you could not see any liquor flowing (Hughes, 2009). Thus, in Willis’s adherence to the ventricular doctrine, there was room for the use of the *thalamus*. Willis’s work was a milestone in brain science and consecrated the use of many anatomical terms, among them the *thalamus*.

## 20th Century: Translation of Arabic Versions of *De Anatomicis Administrationibus*

During the 20th century, the Arabic versions of Galen’s books were translated into modern languages. Max Simon translated into German all books of the Arabic version of *De Anatomicis Administrationibus* (Simon, 1906), and more recently, they have been translated into Italian in an excellent work by Garofalo (1991). In fact, Simon’s modern translation of *De Anatomicis Administrationibus* was the first edition of this book in a Western European language (including Latin) that comprised the fragments where the term *thalamus* appeared.

The Arabic word “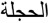 ” (canopy) is used in the printed edition of the last seven books of the Arabic version of *De Anatomicis Administrationibus* by Max Simon. Max Simon translated this word into German as *Schlafzeltähnliche Stelle* [like a bed tent] (Simon, 1906) while Ivan Garofalo translated it into Italian as *alcove* [room] (Garofalo, 1991).

According to Simon (see his note 586), Galen used θαλαμη [thalame] to refer to the portion of the ventricles where the optic nerves originated, with *thalame* being the orifice of a den or cave; and Hunayn used θαλαμζ [*thalamus*], a room for a bride, instead of θαλαμη [*thalame*] (Simon, 1906). Garofalo agreed with Simon (Garofalo, 1991). Did Galen use *thalamus* or *thalame*? Could it be that *thalamus* derives from a misreading of the Arabic versions of Galen’s books that were translated to Latin as suggested (Jones, 2007)? We should recall that Arabic books containing the term *thalamus* were never translated to Latin. Therefore, choosing θαλαμζ [*thalamus*] instead of θαλαμη [*thalame*] and writing it in Latin as *thalamus* could only have been done by the 14th− century translators making the Latin versions from the Greek originals. Actually, Georg Helmreich also used the word *thalame* in his critical edition of the Greek version of *De usu Partium* after collating most of the extant Greek manuscripts of this book and the printed editions of the 16th century (Helmreich, 1907, 1909). However, while the first humanist printed Greek version of *De Usu Partium* in the Aldine edition of Galen’s complete works (Galen, 1525) used θαλαμη [thalame], the first humanist Latin printed edition, the Iuntine edition, used *thalamus* (Galen, 1541). Probably, *thalamus* prevailed over θαλαμη because Latin was the scientific language of those days.

## Conclusion

### Thalamus, an Epic in the History of Anatomy, Medicine, and Science

The preceding paragraphs recount the story of the term *thalamus* from its first use by Galen in the 2nd century A.D. to the 17th century, when it was used by Willis in his influential work on brain anatomy and became an established anatomical term. This eventful story illustrates the complex ways in which anatomy and its terminology originated and were preserved and transmitted throughout history. As an anatomical term, *thalamus* jumped through the centuries from Greek to Syriac, to Arabic, to Latin, from Greek to Latin, and finally, from Latin to the modern languages. It was initially linked to the ventricular doctrine describing a non-existent connection between the brain ventricles and the optic nerves. When anatomists began to question this connection, as well as the most important aspects of the ventricular doctrine in the light of cadaver dissection, Riolan the Younger, a man fascinated by the classical past, again used the term *thalamus*. Willis, another man respectful of tradition, fixed the use of *thalamus* to refer to the ovoid masses on the lateral walls of the third ventricle where the optic nerves originated.

After such a long journey of more than one thousand years from late antiquity to the 17th century, the *thalamus* would still not rest in peace: strong debates on its function and nuclear parcellation roiled through the 19th and 20th centuries leading to the formation of different schools that argued in sometimes bitter debates. But that is another story.

## Author Contributions

MG-C and CC located sources of bibliography including ancient manuscripts, printed books, and facsimile editions. MG-C, IP-S, and CC reviewed the literature. MG-C, IP-S, and CC prepared the figures. MG-C and CC secured funding. All authors contributed to the article and approved the submitted version.

## Conflict of Interest

The authors declare that the research was conducted in the absence of any commercial or financial relationships that could be construed as a potential conflict of interest.

## Publisher’s Note

All claims expressed in this article are solely those of the authors and do not necessarily represent those of their affiliated organizations, or those of the publisher, the editors and the reviewers. Any product that may be evaluated in this article, or claim that may be made by its manufacturer, is not guaranteed or endorsed by the publisher.
